# Schizencephaly as an Unusual Cause of Adult-Onset Epilepsy: A Case Report

**DOI:** 10.7759/cureus.25848

**Published:** 2022-06-11

**Authors:** Arwa Battah, Theodore R DaCosta, Elayna Shanker, Theodore Jr Dacosta, Iyad Farouji

**Affiliations:** 1 Internal Medicine, Saint Michael's Medical Center, Newark, USA; 2 Medical Education, New York Medical College, Valhalla, USA; 3 Gastroenterology and Hepatology, Saint Michael's Medical Center, Newark, USA

**Keywords:** congenital, central nervous system, seizure, epilepsy, schizencephaly

## Abstract

Schizencephaly is a very rare anatomical malformation of the cerebrum characterized by a cleft extending from the cortex to the ventricles. Usually, this disease is diagnosed at a very young age or in early adulthood. Symptoms may vary depending on the site and the size of the malformation. Here, we are describing the unique case of a 21-year-old female, with a past medical history of migraine-type headaches, who presented after the first-onset seizure and was found to have open-lip schizencephaly. She was started on levetiracetam with no complications. In this report, we are trying to describe the proposed etiology and discuss the typical clinical presentation of schizencephaly and compare it to our adult patient who survived childhood without significant cognitive or neurological impairment.

## Introduction

Schizencephaly is an exceedingly rare disorder of neuronal migration characterized by malformation of the cerebrum. The resulting defect leaves a gray-matter-lined cleft in the brain extending from the ependymal surface of the ventricles to the pial surface of the cortex [1]. The incidence of schizencephaly is around 1/1650 patients with epileptic seizures and/or psychomotor retardation or 1.54/100,000 births [2]. Further, the discovery of schizencephaly in adulthood is so rare that the incidence in this demographic has not been published. In this report, we discuss a case of adult-onset open-lip schizencephaly in a 21-year-old female diagnosed after the patient presented with a first-onset seizure. The unique part of our case is the late presentation of the seizures in an adult who had a normal neurological development during childhood and early adulthood. We are reporting this case to increase the awareness of these situations as a cause of adult-onset epilepsy.

## Case presentation

A 21-year-old female with a past medical history of migraine-type headaches presented to the Emergency Department (ED) after an episode of rapid jerking movements, drooling, and profuse sweating witnessed by her friends. There was no tongue biting or bowel/bladder incontinence. She did not remember the episode and was drowsy afterward. She slept through the night but awakened with a headache and non-bilious, non-bloody vomiting, which prompted her visit to the ED. She denied any past seizures or seizure-like activity. At the time of admission, the patient complained of an associated 5/10 persistent frontal headache without radiation, which began after the witnessed seizure. This headache was similar in quality to the migraines she has experienced in the past. She denied any fever, chills, visual changes, numbness, gait changes, depression, and anxiety. When further questioned on her history of migraines, the patient explained that her headaches occur two to three times a week and each headache lasts around 48 to 72 hours. There are no preceding aura symptoms like watery eyes, running nose, or flashes of light. She takes ibuprofen daily (sometimes up to three times a day) with minimal relief.

**Figure 1:**
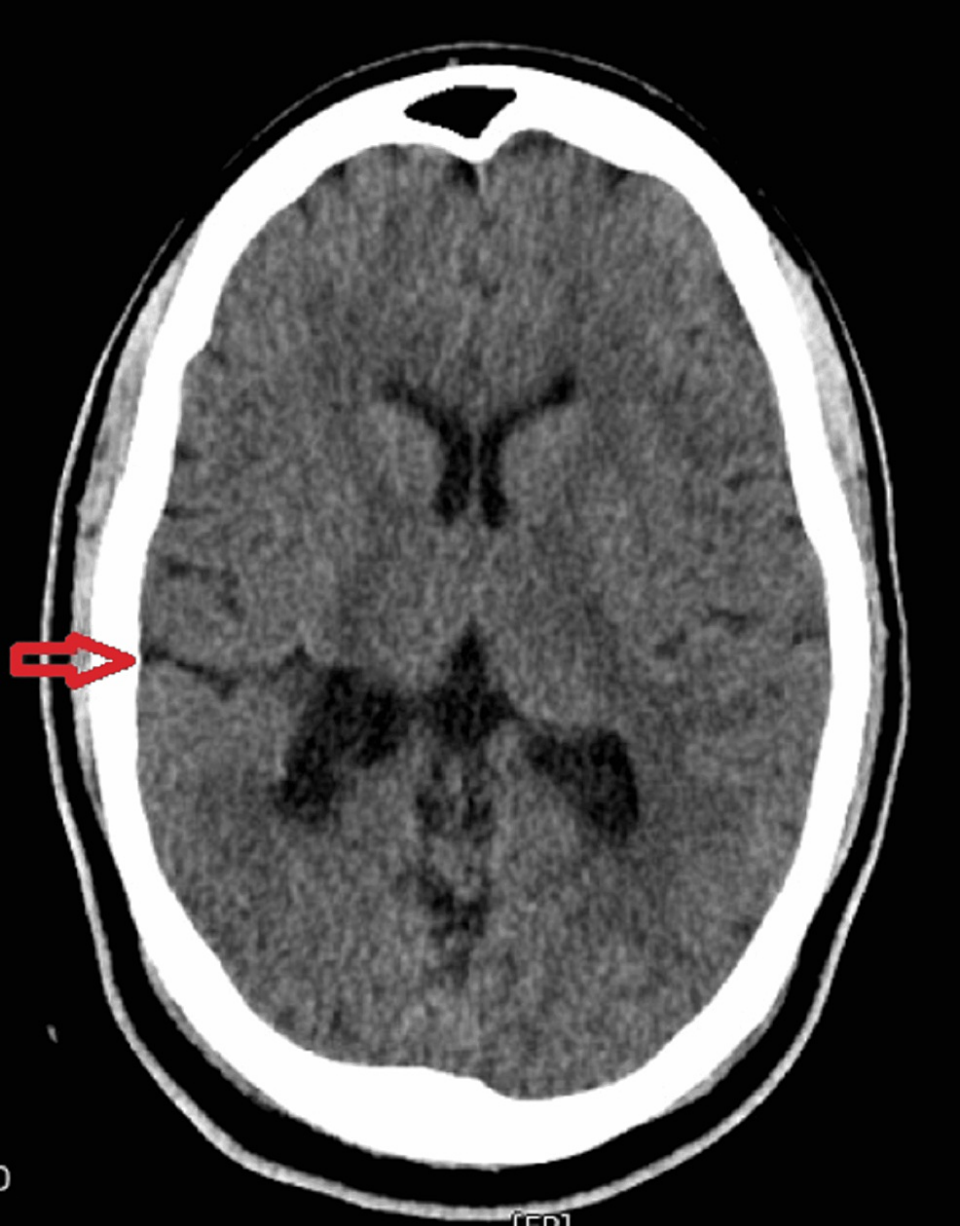
CT of the head without contrast identified open-lip schizencephaly on the right (red arrow) with possible superimposed periventricular leukomalacia adjacent to the right atria.

Family history and birth history were noncontributory. There were no developmental delays during the patient's childhood. An extensive neurologic exam revealed decreased sensation to both pinprick and light sensation at the level of L3-L4 dermatomes of the primal lower extremities, more prominent over the right side. The rest of the workup, including complete blood count and comprehensive metabolic panel, were unremarkable. Computerized tomography (CT) of the head without contrast identified open-lip schizencephaly on the right with possible superimposed periventricular leukomalacia next to the right atria (Figure 1). A 24-hour extended video electroencephalogram (EEG) monitoring revealed asymptomatic right temporal seizure activities. ​​

## Discussion

Schizencephaly is a rare condition of abnormal gyral development resulting from disturbances of neuronal migration in early gestation [1]. Typically, it is diagnosed in infancy or early childhood (before 10 years of age) while working up etiologies for seizure disorders or unexplained neurodevelopmental delays [3]. Schizencephaly may be also diagnosed in prenatal ultrasonography, Less often, but perhaps increasing through the use of in utero MR imaging, schizencephaly is diagnosed even before birth [4].

There are two reported subtypes of schizencephaly, open-lip and closed-lip, with the major difference being that in closed-lip schizencephaly, the opposing walls of the defect are in contact with one another, whereas, in open-lip schizencephaly, the walls do not communicate and are separated by the resulting cleft of cerebrospinal fluid [5]. Open-lip schizencephaly is typically associated with a larger degree of functional impairment [6]. Schizencephaly can be further categorized by location within the cerebral cortex, by laterality (unilateral versus bilateral), or by size (small versus medium versus large) [7].

CT or MRI can diagnose the disease [8]. The imaging shows schizencephaly as a linear cleft lined with heterotrophic gray matter and extends from the cortical surface to the ventricular system. The gray matter within the cleft is dysplastic (polymicrogyria) [8]. The abnormal gray matter in the schizencephaly is not only located in the cleft but also in other areas, even if it is not directly connected to the clefts [9]. The dysplastic gray matter may be located in the contralateral hemisphere in the same or similar location in patients with schizencephaly, forming the so-called mirror focus [10]. Dysplastic gray matter may make up an epileptogenic zone [10].

The clinical presentation of schizencephaly varies significantly. Patients can present with abnormal neurodevelopmental delay, seizure disorder, hemiparesis, or even quadriparesis depending on the size and the site of the malformation [11]. The literature suggests some associations between certain features and prognosis. For example, having bilateral schizencephaly is associated with more profound motor and neurological deficits compared to unilateral defects [12]. Similarly, larger defects or those located involving the frontal lobe tend to carry a harsher prognosis. With so many anatomic variations within schizencephaly, there are understandable differences in case severity with symptoms ranging from new-onset or refractory seizures to hemiparesis and even significant cognitive and neurologic deficits [13].

Interestingly, our patient only sought medical care as a young adult after experiencing a first-onset seizure. Although seizure disorders are the most common initial presentation of schizencephaly patients, these patients typically present before three years of age [14]. The interesting and unique part of our case was that the patient did not complain of any significant motor, cognitive, or social deficits and presented with epilepsy at the age of 21. After extensive workup, the patient was found to have right-sided schizencephaly, which responded well to single-agent anticonvulsant therapy. It is difficult to determine with any certainty why this patient’s case was so mild compared to many others’ lesions that show similar attributes on radiographic imaging.

## Conclusions

Although schizencephaly is rarely diagnosed in adulthood, it is important to recognize the common presentations of the disease. A patient presenting with new-onset seizures or neurological deficits are commonly imaged with a CT scan or MRI, but recognizing these symptoms as signs of a potential malformation such as schizencephaly can help with early diagnoses and adequate treatment to prevent further complications from arising. Understanding the wide range of symptoms in these patients and the likely association with the site and size of the malformation may aid in this process. Through this case, we hope to increase awareness of schizencephaly in adults and the potential for undiagnosed neurological symptoms or new-onset seizures to potentially arise from these malformations. Further research in patients presenting symptomatically at an older age may provide further information on the complexity of this disease and potentially provide the clinical community the information needed for early diagnosis and prevention of complications that can arise if left unrecognized and untreated. The goal of reporting this case is to raise the awareness of this malformation as a serious cause of epilepsy.
